# SLE-diseaseome: a comprehensive meta-collection of systemic lupus erythematosus relevant functional pathways

**DOI:** 10.1093/bioadv/vbag061

**Published:** 2026-02-18

**Authors:** Daniel Toro-Domínguez, Chang Wang, Iván Ellson-Lancho, Jordi Martorell-Marugán, Pedro Carmona-Sáez, Marta E Alarcón-Riquelme, Frédéric Baribaud

**Affiliations:** Unit of Inflammatory Diseases, Department of Environmental Medicine, Karolinska Institute, Solna, 171 77, Sweden; ICV Translational Early Development, Bristol Myers Squibb, Lawrence, NJ 08543, United States; ICV Translational Early Development, Bristol Myers Squibb, Lawrence, NJ 08543, United States; GENYO, Centre for Genomics and Oncological Research: Pfizer, University of Granada, Andalusian Regional Government, PTS Granada, Granada 18016, Spain; GENYO, Centre for Genomics and Oncological Research: Pfizer, University of Granada, Andalusian Regional Government, PTS Granada, Granada 18016, Spain; Fundación para la Investigación Biosanitaria de Andalucía Oriental-Alejandro Otero (FIBAO), Granada 18012, Spain; Fundación para el Fomento de la Investigación Sanitaria y Biomédica de la Comunidad Valenciana (Fisabio), Joint Unit in Biomedical Imaging and Artificial Intelligence FISABIO-CIPF, Valencia 46020, Spain; GENYO, Centre for Genomics and Oncological Research: Pfizer, University of Granada, Andalusian Regional Government, PTS Granada, Granada 18016, Spain; Department of Statistics, University of Granada, Granada 18071, Spain; Unit of Inflammatory Diseases, Department of Environmental Medicine, Karolinska Institute, Solna, 171 77, Sweden; GENYO, Centre for Genomics and Oncological Research: Pfizer, University of Granada, Andalusian Regional Government, PTS Granada, Granada 18016, Spain; ICV Translational Early Development, Bristol Myers Squibb, Lawrence, NJ 08543, United States

## Abstract

**Motivation:**

Systemic lupus erythematosus patients exhibit a broad clinical spectrum of manifestations and suffer from high rates of treatment failure. These can be attributed to disease heterogeneity due to differentially dysregulated pathways. Precision medicine considering the individualized molecular disease driving mechanisms is a promising strategy to address challenges imposed by disease heterogeneity. Available patient blood transcriptome data coupled with pathway-based single-sample scoring approaches have been extensively employed to reveal molecular footprints of disease states and progression as well as delineate population heterogeneity. However, systemic understanding of pathways involved in disease pathogenesis remains lacking.

**Results:**

We created a SLE-diseaseome, an integrative multi-cohort collection of disease-relevant functional gene sets. This resource contains a comprehensive collection of disease-specific gene signatures combining knowledge from several pathway databases and signature sources robustly defined by integrating multiple studies. It offers reliable and extensive reference signatures in a disease-specific manner for functional interpretation of molecular data from clinical studies.

**Availability and implementation:**

The code used to run the pipeline and the R object containing the SLE-diseaseome collection are available at https://github.com/dtordom/SLEDiseaseome.

## 1 Introduction

Systemic lupus erythematosus (SLE) is a complex autoimmune disease characterized by unpredictable patterns of flares and remissions, affecting a wide range of tissues and organs causing significant suffering and mortality. Both the clinical picture and treatment efficacy vary enormously among patients, primarily attributed to the underlying molecular heterogeneity across patients (Allen *et al.* 2021).

Transcriptomics data offer a powerful tool to dissect complex molecular patterns. However, despite significant efforts to untangle the SLE heterogeneity, current knowledge on SLE pathogenesis is still limited to a few well-known and validated pathways, such as those related to type I interferon (IFN-I) (Rönnblom and Leonard 2019). Uncovering this SLE heterogeneity is hindered by intrinsic factors due to disease heterogeneity and extrinsic confounding factors due to batch effects or technical variables. Nevertheless, comprehensive understanding of disease-relevant functional pathways with good sensitivity and reproducibility remains a pivotal task to improve the treatment paradigm for patients and for therapeutic discovery or translational research in SLE.

Multiple pathway databases containing large-scale annotations that link genes to molecular or cellular functions based on evidence from text mining or co-regulation data (Kanehisa *et al.* 2017, 2023, Rinchai *et al.* 2021, Milacic *et al.* 2024) provide the foundation for characterization and identification of disease-related functional pathways. However, cautions should be taken when choosing a particular database for such a task, depending on the type of data included and its potential downstream impact on the functional interpretation. On the other hand, concerns arise when directly deploying multiple databases due to the redundancy in pathways across them. The *MSigDB* project made the effort to integrate multiple pathway databases (Liberzon *et al.* 2015), achieving improved coverage across all biological functions and presenting a strategy for harnessing existing knowledge. However, disease-specific pathways, which are particularly suited for targeted queries in each disease context, remain to be determined.

Many studies have set out to characterize SLE-relevant pathways that delineate disease from healthy states. They reported a broad spectrum of dysregulations in molecular functions and activities (Rai *et al.* 2016, Panousis *et al.* 2019, Wang *et al.* 2024). However, reported pathways or signatures have often not been fully validated across studies or in independent cohorts. Therefore, concerns regarding reproducibility due to potential remnants of cohort-specific signals remain. However, several multi-cohort approaches have been proposed to enable the identification of robust signatures across different studies, while preserving conserved patterns in small subgroups of patients (Toro-Domínguez *et al.* 2022, Hubbard *et al.* 2023). These approaches provide new methodological opportunities to harness public datasets and improve reproducibility in determining disease-specific pathways.

Here, we define a comprehensive collection of SLE-relevant gene signatures, termed the SLE-diseaseome, by implementing a multi-cohort approach, enhancing the capture of disease heterogeneity, and integrating multiple layers of database-derived biological knowledge. The SLE-diseaseome is a SLE-specific knowledge database that can be deployed for applications requiring pathway-level interpretations, and the developed pipeline can be applied to other diseases or biological contexts.

## 2 Methods

### 2.1 SLE datasets and pathway databases

To ensure comprehensive collection of SLE transcriptome datasets, an exhaustive search was performed in the National Center for Biotechnology Information (NCBI) Gene Expression Omnibus (GEO) (Clough *et al.* 2024) database with the following selection criteria: microarray or RNA-seq datasets containing whole blood (WB) or peripheral blood mononuclear cell (PBMC) samples collected from SLE patients—including pediatric or adult age groups—and normal healthy volunteers (NHVs), with at least 10 samples per disease group. Only observational studies or baseline and placebo treated samples from clinical trials were retained. Datasets with SLE and NHV samples from different tissues (e.g. one from WB and the other from PBMC) were excluded. For those containing samples from different tissues in both disease groups or different disease subtypes (e.g. both WB and PBMC from all the disease groups), or profiled on more than one platform, the dataset was stratified by all these factors so that all the SLE and NHV samples in each new dataset remained comparable. Such datasets were then analysed independently. Additionally, SLE data generated in the PRECISESADS project (Barturen *et al.* 2021) was also included. The SLE-diseaseome was generated by incorporating molecular disease mechanisms from 15 blood transcriptome datasets that comprise a total of 5167 SLE and 861 NHV samples. Clinical, demographical, and technical details are available in Supplementary Table S1.

Multiple well-established pathway databases were included as pooled references to survey SLE-specific signatures. These included three immune-related databases of co-expressed gene modules available in the tmod [gene modules from Li *et al.* (2014) and Chaussabel *et al.* (2008)] and BloodGen3Module R packages (Chaussabel *et al.* 2008, Li *et al.* 2014, Weiner and Domaszewska 2016, Rinchai *et al.* 2021), the three categories of gene ontology (GO) (biological process, cellular component, and molecular function) (Aleksander *et al.* 2023), Reactome (Milacic *et al.* 2024), Kyoto Encyclopedia of Genes and Genomes (KEGG) (Kanehisa *et al.* 2023), Wikipathways (Agrawal *et al.* 2024), and blood cell signatures from xCell (Aran *et al.* 2017). Additionally, custom gene sets from previously reported SLE-related signatures, focusing on experimentally validated interferon signatures (Gómez-Bañuelos *et al.* 2024), were also included, comprising a total of 23 676 pathways. Incorporating both immune-specific and general pathway resources ensures comprehensive coverage of biological processes. It is worth noting that MSigDB, another commonly used general pathway database, was not included because most of its collections substantially overlap with databases that have already been included. Further information about pathway resources is available in Supplementary Table S2.

### 2.2 Data pre-processing

**Figure 1:**
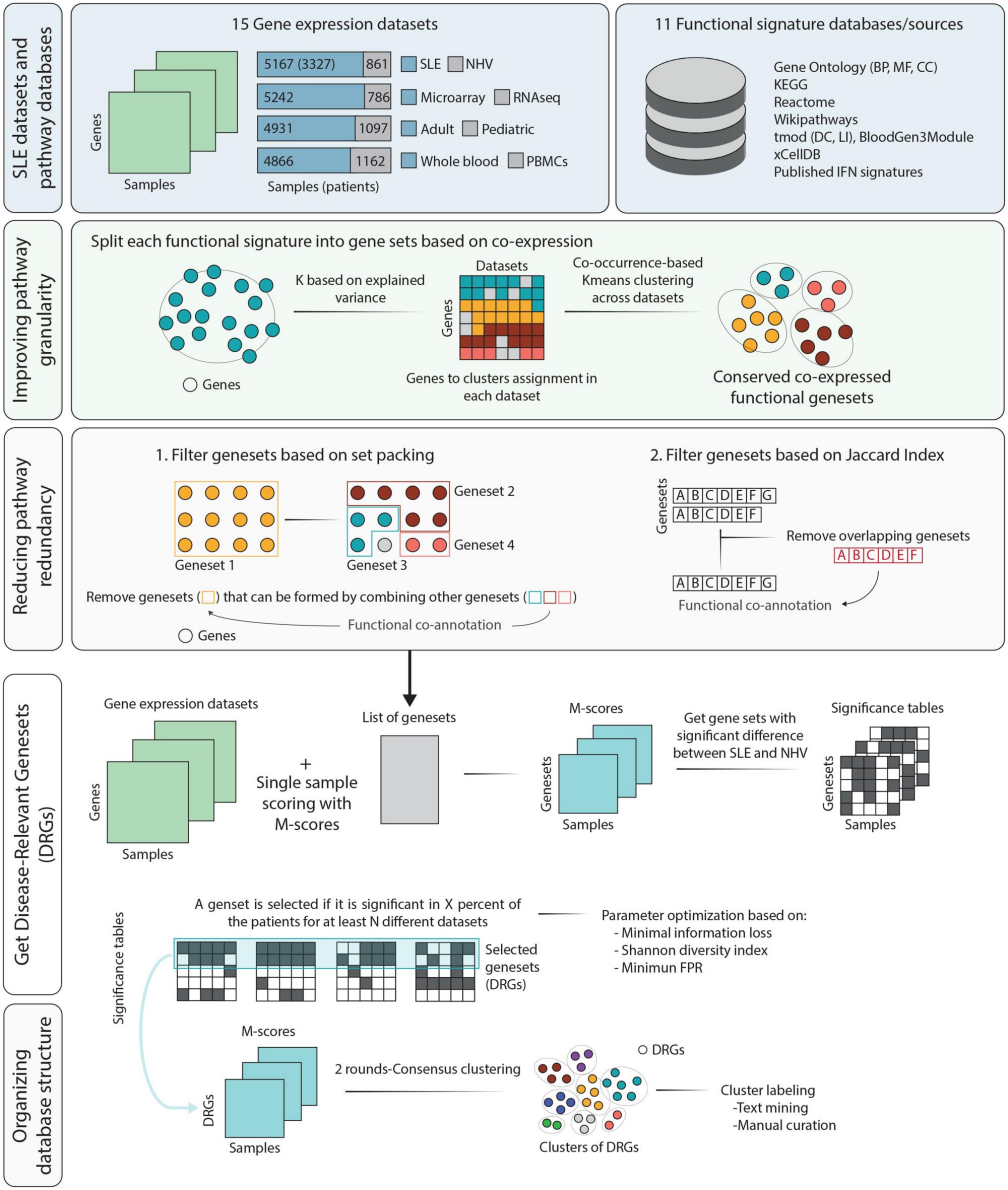
SLE-diseaseome generation pipeline. The workflow shows the datasets and databases used in this study, as well as the four major steps for the generation of the SLE-diseaseome collection, including improvement of the granularity of the biological signatures, redundancy filters, DRGs selection, and the organization of the DRGs into functional clusters.

Transcriptome data was retrieved from various sources. The following datasets: GSE24706, GSE50772, GSE61635, GSE72509, GSE82221, GSE108497, GSE110169, and GSE110174 were downloaded from the Autoimmune Diseases Explorer (ADEx) database (Martorell-Marugán *et al.* 2021); the PRECISESADS dataset was obtained upon request to the authors (Barturen *et al.* 2021); and GSE65391, GSE45291, GSE211700, GSE88887, and GSE22098 were downloaded from NCBI GEO. Each dataset was processed independently in a platform-specific manner as described in ADEx (Martorell-Marugán *et al.* 2021). From raw data to gene expression matrices, the main pre-processing protocols included background correction and quantile normalization (Shi *et al.* 2010) using the *neqc* function from *limma* R package(Ritchie *et al.* 2015) for Illumina microarrays, and probe filtering based on intensity followed by Robust Multichip Average normalization using the *affy* package (Gautier *et al.* 2004) for Affymetrix platforms. For RNA-seq data, we started from count matrices rather than raw FASTQ files due to data availability, and subsequently genes with zero counts in more than 10% of the samples were discarded and counts were normalized using the trimmed mean of *M*-values (TMM) method available in the *NOISeq* R package (Tarazona *et al.* 2015). The gene expression matrices from RNA-seq or microarray platforms were subjected to logarithmic transformation, followed by annotation of the transcripts into gene symbols. Duplicated genes were merged by taking the median expression level. Genes with zero or close to zero variance (i.e. standard deviation < 0.05) in each disease group (i.e. NHV or SLE) were filtered out using the caret R package (Kuhn 2008). The pipeline to generate the full diseaseome resource involves four further major steps summarized in Fig. 1.

### 2.3 PathMED-based analytical workflow

PathMED is an R/Bioconductor package designed to provide a unified framework for the analysis of omics data in the context of precision medicine (“pathMED,” n.d.). The diseaseome pipeline employs several core functions implemented in the PathMED package at different steps. First, the *dissectDB* function enables the refinement of functional annotations by increasing pathway granularity, subdividing broad biological pathways into smaller, co-expressed gene sets with higher functional resolution. Second, the *getScores* function transforms gene-level expression data into pathway or gene-set activity profiles using single-sample scoring approaches, allowing selection from among 20 different scoring methods. Third, the *mScores_filterPaths* function was used to identify disease-relevant gene sets (DRGs) through a subset-based meta-analysis framework. It enables the selection of gene sets that are strongly dysregulated in specific patient subgroups relative to NHVs, while ensuring robust detection across multiple independent studies. Finally, the *trainModel* function leverages gene-set activity matrices to train and evaluate generalizable predictive models for clinical variables of interest. Together, these components provide a flexible and modular framework to transform high-dimensional molecular data into biologically interpretable and clinically actionable features.

### 2.4 Improving pathway granularity and specificity

Pathway annotations often reflect broad associations among a large number of gene members, which in most cases are defined by different criteria according to the database, regardless of the biological context. This could result in generic biological pathway annotations, comprised of genes with different behaviors. To increase the granularity and specificity of the annotations, pathways from the original pathway databases were split into gene sets based on gene co-expression patterns for a given pathway across multiple SLE datasets. We considered that a biological pathway must contain at least three genes to be meaningfully analyaed, as many single-sample scoring and enrichment methods require a minimum of three features to compute reliable statistics and to avoid results being driven by single genes. The *dissectDB* function from *pathMED* R package (“pathMED,” n.d.) was used, with the following parameters enabled: the minimum number of genes in a pathway for splitting (*mimPathSize*) was set to 8, the minimum number of genes in a resulting gene set (*minSplitSize*) was set to 3, the maximum number of resulting gene sets (*maxSplits*) was set to NULL to remove any restrictions, and the minimum percentage of the cumulative explained variance of a given pathway (*explainedVariance*) was set to 70 for the selection of the number of gene clusters for the corresponding pathway. Details regarding the selection of parameter values are provided in Supplementary File S1.

For the dissectDB function, two types of lists were provided as input, one list with gene expression matrices from 15 SLE datasets, and the second list with a total of 23 676 pathways pooled across 11 different pathway sources as described above. Specifically, the following steps implemented in the *dissectDB* function were run for each pathway that met the requirement set in *mimPathSize*. For each dataset, *z*-scores of each gene in the SLE samples were compared with the distribution in NHVs to quantify the level of dysregulation at the gene level. Next, the number of gene clusters was determined with the cumulative explained variance set in *explainedVariance* and genes were assigned to the clusters by *k*-means based clustering of each dataset, using Euclidean distance. Using *z*-scores as input for *k*-means clustering enables the capture of both the magnitude and direction of gene expression dysregulation relative to NHVs. Consequently, genes that are from the same pathway but exhibited consistently opposite regulation patterns across SLE samples can be effectively separated into clusters. Finally, subsequent *k*-means-based co-occurrence clustering across datasets was performed to define the clusters of genes (gene sets) within a pathway, using a matrix of gene cluster assignments at the dataset level as input, where rows represented the genes and columns represented the datasets. When applicable, gene clusters (gene sets) with the number of genes below the limit set in *minSplitSize* were merged with the nearest cluster based on their Euclidean distance. Among the 47 337 gene sets obtained, those with less than three genes were further removed, resulting in a total of 38 797 gene sets for further processing.

### 2.5 Reducing pathway redundancy

Pathway redundancy can arise when integrating multiple pathway databases, resulting in similar pathways with different functional annotations, or after splitting large pathways into smaller composite gene sets as described above, resulting in overlap among them. To mitigate redundancy, two filtering approaches were applied to all the gene sets based on the set packing algorithm for combinatorial optimization and the similarity-based Jaccard index metric.

To implement the set packing algorithm, first, for each gene set *G*, smaller gene sets {*g*_1_, *g*_2_, …, *g*_*n*_}, which were subsets of *G* (*g*_*i*_ ⊆ *G*) (Stoney *et al.* 2018), were identified from the collection of split gene sets. The optimal combination of small gene sets was selected based on their combined coverage of the genes in *G* to achieve the largest overlap. Then, two metrics—Pearson correlation coefficients and information gain—were computed based on single-sample gene-set scores (i.e. *M*-scores (Toro-Domínguez *et al.* 2022)) and used to determine the retention of *G* or the collection of selected *g*_*i*_s. Specifically, *M*-scores were calculated with all SLE datasets in relative to the distribution in NHVs from the corresponding dataset to assess the dysregulation of gene sets *G* and selected *g*_*i*_s at the individual patient level. The average Pearson correlation coefficients were calculated between *M*-scores of *G* and *g*_*i*_s across datasets. The information gain was defined as the average difference in the proportion of patients with significant *M*-scores (∣*M*-score∣ ≥ 1.65, equivalent to *P*-value ≤.05 in a normal distribution) between *G* and selected *g*_*i*_s across datasets to reveal their abilities to measure gene-set dysregulation. If the average correlation coefficient was larger than 0.75 and the average information gain was less than 10%, the large gene set *G* was retained, while all the small gene sets {*g*_1_, *g*_2_, …, *g*_*n*_} were removed, indicating that small gene sets failed to fully replace the large gene set *G*. Otherwise, if the information gain was greater than 10%, the selected small gene sets *g*_*i*_s were retained, while *G* and other small gene sets were removed. Co-annotation of the retained gene set(s) was performed by merging their functional terms with those from the removed gene sets to incorporate all relevant functional annotations.

Next, a similarity matrix was constructed using Jaccard index comparisons of retained gene sets. Pairs of gene sets with Jaccard indexes greater than 0.8 (or 0.75 for gene sets with less than five genes) were identified using the *graph_from_adjacency_matrix* function in the *igraph* R package (Csárdi *et al.* 2025). Within each group of highly similar gene sets, the largest one was further retained and re-annotated by adding the functional annotations of the other gene sets in the group to its original term, while excluding the rest from further analysis.

Both filters resulted in a total of 36 853 gene sets. Together, these first two steps improved the resolution of functional annotations, especially for large pathways, and enabled pinpointing specific branches of a pathway for follow-up interrogation or downstream development of biomarker panels.

### 2.6 Identifying SLE-related gene sets

The next step in the pipeline was designed to identify DRGs, which allowed to focus on molecular mechanisms of SLE instead of pan-biological signatures. This was achieved by implementing the strategy previously described by us (Toro-Domínguez *et al.* 2022), which enabled the identification of consistent molecular signatures in subgroups of patients across cohorts. To do this, the *mScores_filterPaths* function in the *pathMED* R package (“pathMED,” n.d.) was used, accounting for disease heterogeneity while avoiding dataset-specific signals (Toro-Domínguez *et al.* 2022). Specifically, instead of focusing only on DRGs present in all patients, this function enabled the detection of DRGs from groups of patients when comparing with NHVs, while requiring consistent detection across multiple studies to eliminate results that could be driven by single datasets. To accomplish this, the function required the optimization of two parameters: *perc_samples*, which represents the minimum percentage of patient samples significantly dysregulated in a dataset when compared to NHVs (∣*M*-score∣ ≥ 1.65, equivalent to a *P*-value ≤.05 in a normal distribution) from the corresponding dataset, and *min_datasets*, which specifies the minimum number of datasets where a gene set needs to be significant in order to be considered as conserved across datasets. Fine tuning of those parameters is important, as lower values can lead to more permissive analyses with higher false-positive rates, while higher values can result in the loss of true signals from smaller patient groups. To optimize the parameters, a set of 1000 random gene sets was created, with sizes ranging from the 10th to the 90th percentiles of the sizes of all the gene sets obtained from previous steps. This served as a technical background under the assumption that there were no significant differences in their scores when comparing with NHVs. After calculating the *M*-scores with those testing gene sets, three metrics were computed to determine the optimal combination of parameters. First, the false-positive rate (FPR)—defined as the number of significant random gene sets (∣*M*-score∣ ≥ 1.65)—was calculated when using each combination of *perc_samples* and *min_datasets* parameters (ranging from 1 to 30 and 1 to 15, respectively). Second, information loss in each dataset was evaluated. With each parameter combination, the number of principal components required to explain 80% of the cumulative variance across patient samples when using all the gene sets was compared with the number required when using the filtered gene sets. The average information loss across all the datasets was calculated and considered together with other metrics for parameter value selection. Lastly, to quantify the functional diversity, the Shannon index was computed for the roots of selected functional terms. Taken together all the metrics, a step-wise filtering was implemented across all the parameter combinations to select the one with best performance, which resulted in significances in at least 10% of the samples and 30% of the datasets, an FPR of less than 5%, a minimal average information loss and a higher Shannon index. Based on all these considerations, DRGs were defined as gene sets that were significantly dysregulated in at least 12% of patients when compared to the NHVs in each dataset and consistently detected in seven out of the 15 datasets (Supplementary Fig. S1), resulting in a total of 5212 DRGs, comprising 9830 unique genes, from blood retained in the SLE-diseaseome.

When analysing the influence of different collections on the final selection of DRGs, we observed that the initial splitting substantially increased pathway granularity across all databases, particularly for large canonical pathway resources such as KEGG, Reactome, and WikiPathways (Supplementary Tables S2 and S3). Importantly, subsequent redundancy reduction led to only modest decrease in pathway numbers, indicating that most gene sets possess distinct gene composition patterns. These results also suggest that the similarity criteria applied were intentionally stringent, assigning only highly overlapping pathways as redundant. Additionally, DRGs were consistently enriched in immune-focused and data-driven resources, including BloodGen3Modules, tmod and xCell, as well as literature-derived signatures previously associated with the disease (Supplementary Table S3). In contrast, general resources such as Gene Ontology and Reactome provided extensive biological coverage, albeit with lower relative enrichment. Together, these results support the complementarity of general and disease-focused databases, underscoring the importance of integrating multiple functional resources to construct a comprehensive, disease-specific diseaseome.

### 2.7 Clustering SLE-related gene sets

To integrate functional annotation terms across different databases and facilitate the interpretation of results derived from the SLE-diseaseome, a three-level functional labeling approach was implemented. First, the *M*-scores of the SLE-diseaseome gene sets calculated from all the datasets were combined into a single matrix used for an initial round of clustering (round 1). The optimal number of gene-set clusters was determined using the *M3C* and *NbClust* R package (Charrad *et al.* 2014, John *et al.* 2020) with 1000 iterations, following a multi-step strategy. Specifically, M3C was first used to identify statistically supported candidate *K* values that demonstrated evidence of nonrandom clustering structure. Subsequently, NbClust was applied to rank these candidate *K* values based on cluster stability across all internal validation indices available in the package. Final cluster assignment was then performed using *k*-means-based consensus clustering implemented in the *ConsensusClusterPlus* function in the *ConsensusClusterPlus* package (Wilkerson and Hayes 2010), with 500 permutations and complete linkage. The final choice of *K* was guided by both statistical support and biological interpretability, favoring solutions that grouped functionally or semantically related gene sets while avoiding overly small clusters or those containing only a single gene set.

This step identified 32 clusters of gene sets with similar patterns of gene-set dysregulation across datasets, which correspond to main functional categories. Next, a second round of clustering was performed within each gene-set cluster independently for further stratification (round 2). To enable the identification of more detailed patterns for each initial cluster, the optimal number of subclusters was calculated each time, resulting in a total of 236 clusters that reflected the combined number of subclusters derived from the initial 32 clusters. Lastly, new functional labels were assigned to each cluster in each round of clustering based on the original functional terms of gene sets within the cluster. This step was done by combining text mining implemented in the tm R package (Zondervan and Tolentino-Zondervan 2023), which identified the most representative roots and keywords among a group of terms, followed by manual curation to ensure accurate and optimal representation of the biological and functional context of each cluster. With this strategy, the SLE-diseaseome collection was organized into three distinct biological levels: (1) 32 broad main functions derived from the initial clustering (round 1), (2) 236 specific functions within each main function derived from the second round of clustering (round 2), and (3) 5212 functional terms at the individual gene-set level. Details can be found in Supplementary Table S4.

## 3 Results

### 3.1 User case 1: associating gene sets with clinical features

**Figure 2:**
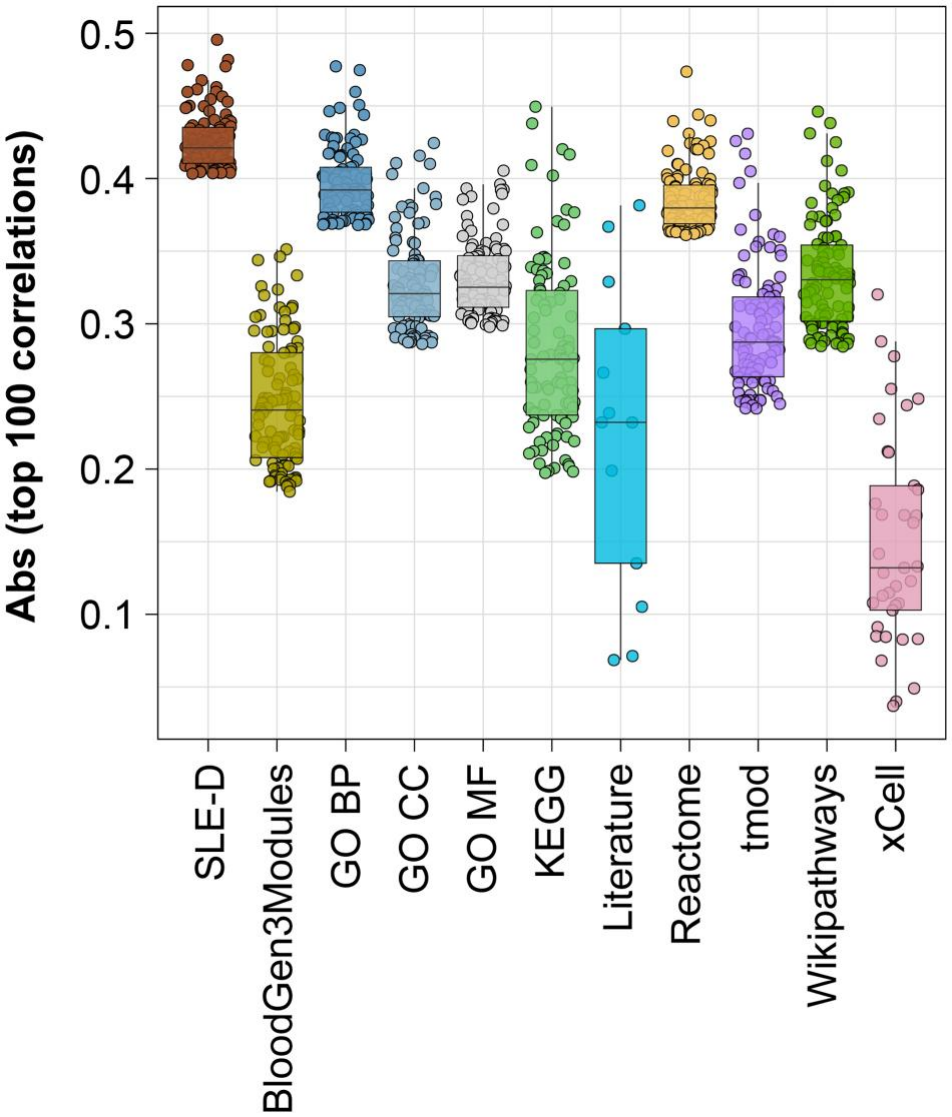
Comparison of correlations between SLEDAI and gene-set scores across different pathway collections. Box plot showing Pearson’s correlations for the top 100 gene sets with the highest absolute correlation coefficients from each pathway resource. SLE-D: SLE-diseaseome; GO BP: gene ontology (biological process); GO MF: gene ontology (molecular function); GO CC: gene ontology (cellular component).

To demonstrate how our curated gene-set collection, with its increased functional granularity, more accurately reflects clinically relevant phenotypes compared to existing pathway resources, we assessed the association between gene-set scores and clinical disease activity measured by the Systemic Lupus Erythematosus Disease Activity Index (SLEDAI), a composite score widely used to summarize overall disease activity in SLE. We evaluated the correlation between gene-set *M*-scores derived from each pathway resource, including SLE-diseaseome, and SLEDAI scores using four datasets (i.e. PRECISESADs, GSE65391, GSE45291, and GSE88887). Figure 2 summarizes the Pearson’s correlations of the top 100 gene sets with the highest absolute correlation coefficients within each resource. Gene sets from the SLE-diseaseome consistently showed stronger and more significant correlations with SLEDAI than those from other pathway databases. These results indicate that the increased granularity of the SLE-diseaseome enables more sensitive detection of clinically relevant molecular patterns. Overall correlation magnitudes were moderate across all resources, likely reflecting the intrinsic heterogeneity and composite nature of the SLEDAI score (Thanou *et al.* 2021).

### 3.2 User case 2: using SLE-diseaseome for molecular stratification of patients

The performance of the SLE-diseaseome to reveal disease heterogeneity through uncovering endotypes and enabling association between molecular endotypes and clinical responses was assessed. It was applied to a blood transcriptome dataset collected from 284 SLE patients, who participated in the phase 2b clinical trial of iberdomide (NCT03161483) (Celgene 2023, Bachali *et al.* 2025) to characterize the disease heterogeneity at baseline. The dataset contains gene expression data collected at baseline and week 24 from subjects who received one of three doses of iberdomide (0.15, 0.3, or 0.45 mg/day) administered once daily or placebo for 24 weeks and processed as described in a previous report (Bachali *et al.* 2025). Treatment response was defined by the SLE Responder Index 4 (SRI-4) (Luijten *et al.* 2012) at week 24, which was the primary endpoint of the trial.

**Figure 3:**
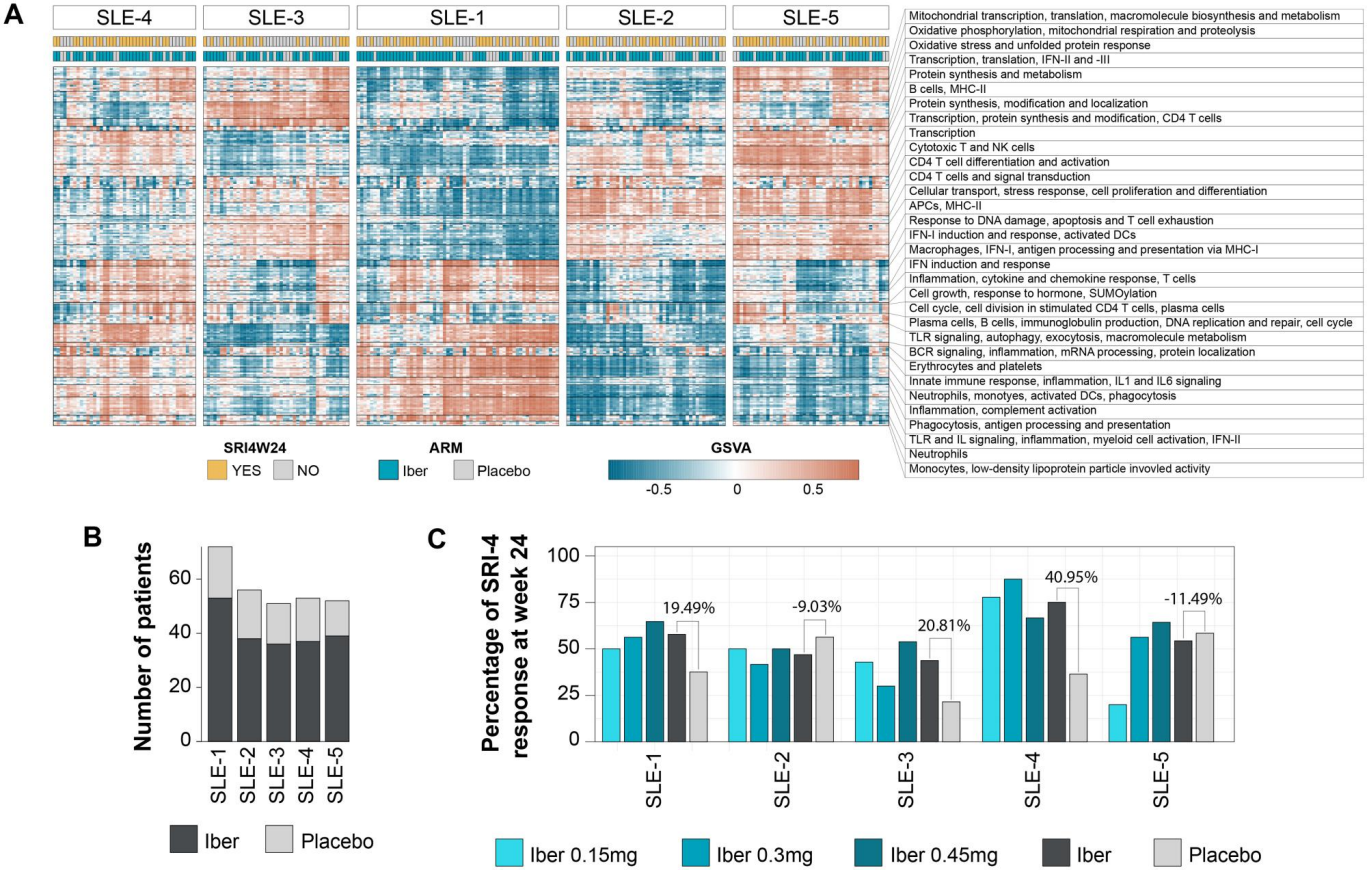
Response to iberdomide in relation to the molecular stratification of SLE patients at baseline. (A) Molecular clustering of patients (columns) using GSVA scores against all the DRGs in the SLE-diseaseome. Rows represent the 236 clusters of DRGs from round 2 clustering. Heatmap colors indicated average GSVA scores from round 2 DRG clusters. Color bars above the heatmap show treatment arms and SRI-4 responses at week 24 [i.e. ARM as placebo or iber (pooled iberdomide doses)]. Patient clusters are indexed as SLE-1 to SLE-5 and then sorted by differences in the SRI-4 response rate between ARMs in a descending order. (B) Number of patients who received either Iber or PBO across patient clusters. Colors denote Iber or placebo treatment. (C) Percentage of SRI-4 response at week 24 (SRI4W24) in each patient cluster by dose and treatment arm. Colors denote treatment groups. The numbers indicate the difference in percentages of the response between the pooled iberdomide and placebo groups. SRI-4: Systemic Lupus Erythematosus Responder Index; ARM: all participants receive the same experimental treatment.

For patient stratification, baseline transcriptome profiles were log2-transformed and TMM-normalized. Gene-set variation analysis (GSVA) enrichment scores (ES) were calculated against DRGs in the SLE-diseaseome using getScores function from pathMED R package (“pathMED,” n.d.). Then, the optimal number of patient clusters was determined using the M3C and NbClust packages (Charrad *et al.* 2014, John *et al.* 2020) with 1000 iterations. Patient clustering was subsequently carried out with *k*-means based consensus clustering implemented with the *ConsensusClusterPlus* function in the ConsensusClusterPlus package (Wilkerson and Hayes 2010), enabling 500 permutations and complete linkage. Five molecularly distinct patient clusters were obtained (Fig. 3A) and comprised of patients from the placebo and iberdomide treatment arms (Fig. 3B). Lastly, the percentage of responders determined based on the SRI-4 at week 24—the primary end point used in the trial—was assessed within each cluster, by comparing each iberdomide dose separately or in combination with the placebo arm. One patient cluster showed a consistently higher response rate across all three doses of iberdomide tested when compared with the placebo (PBO) arm (Fig. 3C). Indeed, the SLE-4 cluster achieved a 75% response rate in patients treated with iberdomide, representing a 40% higher SRI-4 response rate than those who received PBO (Fig. 3C). This cluster showed higher signature scores relative to the other clusters in the cohort (i.e. GSVA score >0) associated with, e.g. inflammation, cytokine response, and IFN-I signaling pathway, and reduced signature scores (i.e. GSVA score <0) in, e.g. B cell and T cell differentiation (Fig. 3A). This finding is consistent with previous analysis performed with 32 gene modules (Bachali *et al.* 2025) suggesting that there are subgroups of SLE patients who might more likely benefit from iberdomide treatment. Furthermore, using the SLE-diseaseome, additional signatures associated with high- or low-responder clusters were revealed, including elevated baseline profile involving, e.g. autophagy, particularly related to macrophages, antigen processing and presentation via major histocompatibility complex I (MHC-I), cell cycle in plasma cells, and immunoglobulin production in the high-responder cluster, as well as higher baseline activity in CD4+ T cell differentiation and activation in the low-responder clusters (Fig. 3A). Overall, this observation demonstrates that the SLE-diseaseome in combination with single-sample gene set scoring can enable molecular stratification, particularly the identification of responder-enriched subgroups, and provide granular and specific gene sets for downstream mining with easy functional interpretations of the results. Finally, it also enables a precision medicine approach to enrich iberdomide responders and identifies the therapeutic mechanisms of action best suited for the patients showing no clinical response to iberdomide.

### 3.3 User case 3: using the SLE-diseaseome for functional enrichment analysis

**Figure 4:**
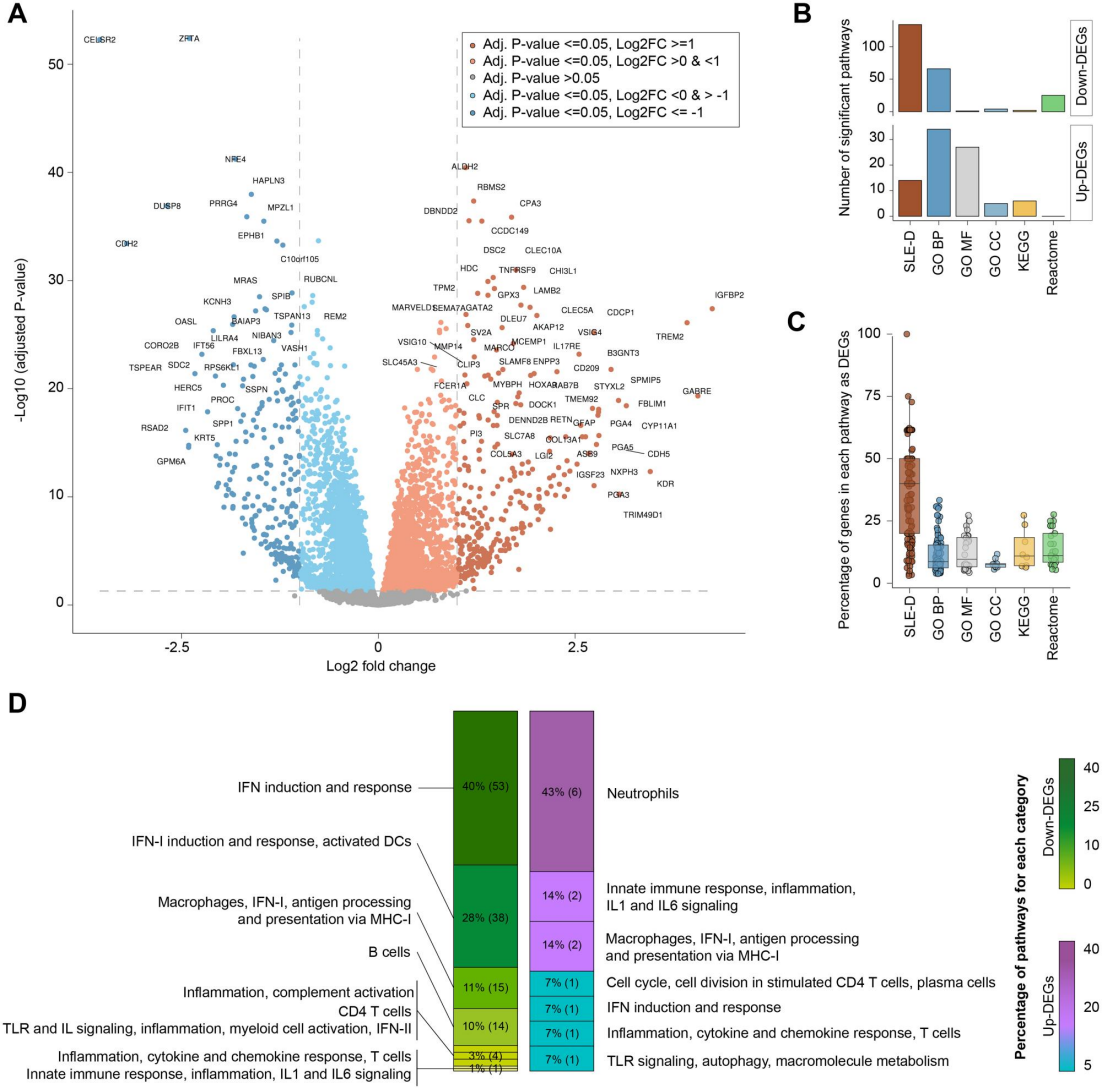
Functional analysis of longitudinal iberdomide effects. (A) DEGs between iberdomide and placebo treated subjects at week 24 controlling for baseline profiles. Colors indicate genes which meet different criteria of adjusted *P*-values and log2(FC). (B) Number of significant pathways obtained from functional enrichment analysis with different pathway databases using up- or down-regulated DEGs. (C) Percentage of hits in significantly enriched gene sets that were also DEGs. (D) Proportion of significant gene sets associated with up- or down-regulated DEGs using the SLE-diseaseome. The gene-set terms are grouped based on their round 1 labels. Color gradients indicate the percentage of functional terms associated with DRGs grouped into each round 1 label among the total number of significant functional terms, which is also indicated on the bars, while the numbers in parentheses indicate the actual counts. SLE-D: SLE-diseaseome; GO BP: gene ontology (biological process); GO MF: gene ontology (molecular function); GO CC: gene ontology (cellular component).

To demonstrate the usability of the SLE-diseaseome for functional enrichment analysis, a common step following differential gene expression analysis, the same iberdomide blood transcriptome dataset was used. Differentially expressed genes (DEGs) comparing iberdomide and placebo treated subjects at week 24 controlling for baseline profiles were determined using the edgeR and variancePartition R packages (Robinson *et al.* 2010, Hoffman and Schadt 2016). Specifically, the model tested for the combined timepoint (i.e. baseline or week 24) and treatment group (i.e. iberdomide or placebo) factors, while accounting for differences in baseline disease activity and corticosteroid dose levels as well as technical factors in data generation and accommodating repeated measures. The contrast was formed as (iberdomide at week 24—iberdomide at baseline)—(placebo at week 24—placebo at baseline) to identify the drug-specific gene expression changes over time. Genes with a Bonferroni corrected *P*-value <0.05 and an absolute log2-fold change >1 were retained as DEGs. Specifically, 292 significantly up-regulated and 238 down-regulated genes were obtained comparing all iberdomide treated patients with patients treated with PBO at week 24, while controlling for baseline profiles (Fig. 4A).

Then, functional enrichment analysis was done using clusterProfiler R package and the different gene-set databases, comparing the SLE-diseaseome to KEGG, Reactome, as well as Gene Ontology biological process (GO BP), the molecular function (GO MF), and the cellular component (GO CC). Significant functional terms with an FDR <0.05 were grouped based on their round 1 annotation labels when using the SLE-diseaseome or new functional labels created in a similar way as the SLE-diseaseome when using other databases to facilitate the interpretation of the results. Among all resources tested, only GO BP and the SLE-diseaseome retrieved more than 10 functional terms with both up- and down-regulated genes, respectively (Fig. 4B), proving their improved ability to associate genes with molecular functions. Higher percentages of hits in gene sets with significant enrichment were observed with the SLE-diseaseome, with an approximately 30% difference on average comparing to all the other resources (Fig. 4C). Additionally, the functional terms retrieved from the SLE-diseaseome clearly indicated major molecular signatures with coherent functional themes, such as neutrophil signatures enriched in up-regulated genes as well as IFN induction and response signatures enriched in down-regulated genes (Fig. 4D). Although some of these functions, particularly the down-regulated IFN response, were also detected with GO BP (Supplementary Fig. S2), SLE-diseaseome enabled the detection of additional terms relevant to the disease, such as B cell related processes.

### 3.4 Generating diseaseome for other diseases

The pipeline and code provided in this study enables the generation of new diseaseomes for other diseases or biological contexts. The proposed pipeline is operable starting with a single omics dataset containing at least three disease samples and reference samples (typically NHVs), together with at least one functional annotation database or resource, although it can also function with a single pathway. Given all required input files, the script 03_RunDiseaseome.R performs every steps necessary for diseaseome construction, including enhancing pathway granularity, reducing redundancy, and selecting DRGs.

Although the pipeline’s minimal requirements are intentionally low, multiple factors affect the robustness and interpretability of the resulting diseaseome collection. For example, including more independent studies may increase the stability and reproducibility of the identified DRGs (with at least two independent studies being a reasonable criterion), whereas incorporating multiple functional annotation resources can enhance biological coverage. Importantly, only pathways consistently associated with the disease are retained in the final diseaseome as DRGs. Therefore, incorporating more pathway databases—even those not disease-specific—should not negatively impact result quality, though it may increase computational costs. In addition, the pipeline is not limited to transcriptomic data and can be applied to other omics data types, such as proteomics, as long as relevant functional pathway annotations can be mapped to the measured molecular entities.

## 4 Conclusions

In this work, we developed the SLE-diseaseome, a comprehensive collection of gene sets with dysregulated expression patterns in SLE, through integration of multiple pathway databases and gene expression datasets. With the pathway splitting strategy, those gene sets represent unique granular functional units relevant to disease pathobiology, providing pinpoint gene lists for precise functional interpretation. The SLE-diseaseome also captures robust and broad-spectrum disease signatures through the integration of multiple expression datasets, increasing reproducibility and reliability. The layout of this collection, with hierarchical annotations based on co-expression, enables more informative and flexible extraction of functional labels and facilitates downstream interpretation in a contextualized manner. Importantly, the SLE-diseaseome provides a unified set of gene sets whose activity can be consistently quantified and compared across independent studies. This enables the identification of disease-, phenotype-, or drug response—associated functions and their core gene components, as well as the direct validation of these functional signatures in external datasets. Moreover, given that patient stratification analyses are highly dependent on the choice of features, the use of a common disease-specific functional reference such as the SLE-diseaseome can facilitate the extrapolation and comparison of molecular endotypes across studies. This collection can be applied to many analyses that require gene-set annotations, including conventional gene-set enrichment and single-sample gene set scoring analyses. Indeed, as demonstrated by the two use cases, the SLE-diseaseome improved granularity and specificity, rendering it particularly suited for patient stratification and functional interpretation of gene targets. It is important to note that this work has a primarily methodological scope and does not aim to establish or validate clinical models in SLE. More extensive studies spanning the full spectrum of clinical manifestations and outcomes, together with large-scale external validation efforts, would be of great interest for future research. The rationale and pipeline implemented in this study to generate a disease-specific gene-set collection can also be readily applied to other diseases or biological contexts.

## Supplementary Material

vbag061_Supplementary_Data

## Data Availability

The SLE datasets used for the SLE-Diseaseome construction, except for the PRECISESADs dataset, can be downloaded from the NCBI GEO database under accession numbers GSE65391, GSE45291, GSE211700, GSE22098, GSE88887 GSE24706, GSE50772, GSE61635, GSE72509, GSE82221, GSE108497, GSE110169, and GSE110174. The last eight datasets mentioned above can also be downloaded in a pre-processed version directly from the ADEx database (Martorell-Marugán *et al.* 2021). The PRECISESADs dataset is available upon request (Barturen *et al.* 2021). The code used to run the pipeline and the R object containing the SLE-diseaseome collection are available at https://github.com/dtordom/SLEDiseaseome. The iberdomide dataset can be obtained upon request, following Bristol Myers Squibb (BMS) policy on data sharing, which may be found at https://www.bms.com/researchers-and-partners/independent-research/data-sharing-request-process.html.
