# Supplementary material for: SLE-diseaseome: a comprehensive meta-collection of systemic lupus erythematosus relevant functional pathways
Source: Bioinform Adv. 2026 Feb 18;6(1):vbag061. doi: 10.1093/bioadv/vbag061 (PMC12989159; doi:10.1093/bioadv/vbag061)
Supplement: vbag061_Supplementary_Data [file vbag061_supplementary_data.zip › SupplementaryTable1.pdf]

Supplementary Table 1: SLE transcriptome datasets used to generate SLE-diseaseome collection. The table records the dataset identifiers, the number of SLE and NHV samples, the SLEDAI and their age group (i.e., pediatric or adult) showing age means and standard deviations by study, the tissue type, region, the sequencing platform, the number of patients by sex and race and additionally relevant clinical or technical information if available. F: female; M: male; AS: Asian; AA: African American; W: White/ Caucasian; HS: Hispanic; AI: American native Indian; O: other; EU: Europe; USA: United States of America; NA: Non available information.

Dataset	Sample size (SLE / NHV)	Tissue	Age	SLEDAI mean (sd)	Sex (F / M)	Race	Region	Platform	Clinical information
PRECISESADS	376 / 263	WB	Adult: 45.82 (±13.69)	6.05 (±5.55)	349 / 27	AS: 3, AA: 4, W: 363, O: 6	EU	GPL16791	A cross-sectional cohort of clinically heterogeneous patients
GSE65391	924 / 72	PBMC	Pediatric: 14.7 (±2.77)	6.28 (±4.59)	137 / 21	AA: 49, W: 33, HS: 76	USA	GPL10558	A longitudinal cohort of clinically heterogeneous patients
GSE45291	727 / 20	WB	Adult: 29.97 (±12.32)	2.1 (±2.35)	275 / 26	AS: 9, AA: 106, W: 175, O: 11	USA	GPL13158	A longitudinal cohort of clinically heterogeneous patients
GSE24706	15 / 33	WB	Adult: NA	NA	NA	NA	USA	GPL6884	Patients with different antinuclear antibody titers
GSE50772	61 / 20	PBMC	Adult: NA	NA	NA	NA	USA	GPL570	Clinical information not available
GSE61635	79 / 30	WB	Adult: NA	NA	NA	NA	NA	GPL570	Anti-ribonucleoprotein positive patients
GSE72509	99 / 18	WB	Adult: NA	NA	NA	NA	USA	GPL16791	Patients with different anti-Ro titers
GSE82221	30 / 25	PBMC	Adult: 30.49 (±8.63)	NA	26 / 4	AS: 30	China	GPL10558	Patients with or without lupus nephritis
GSE108497	325 / 187	WB	Adult: 30.6 (±4.76)	NA	325 / 0	AS: 54, AA: 66, W: 330, HS: 16, O: 13	USA	GPL10558	Study of SLE Patients During Pregnancy
GSE110169	82 / 77	WB	Adult: NA	NA	69 / 13	NA	USA	GPL13667	Clinical information not available
GSE110174	144 / 10	WB	Adult: NA	NA	NA	NA	USA	GPL13158	Baseline SLE samples from patients enrolled in a clinical trial
GSE211700	20 / 10	PBMC	Adult: NA	NA	NA	AS: 20	China	GPL20795	Patients with or without lupus nephritis
GSE22098	82 / 19	WB	Pediatric: 14.85 (±2.77)	NA	69 / 13	AS: 4, AA: 19, W: 12, HS: 47	USA	GPL6947	Clinical information not available
GSE22098	28 / 17	WB	Adult: 38.5 (±13.29)	NA	24 / 4	AS: 2, AA: 6, W: 15, HS: 5	USA	GPL6947	Clinical information not available
GSE88887	2175 / 60	WB	Adult: 41.63 (±12.12)	10.37 (±3.75)	1754 / 141	AS: 21, AA: 251, W: 1322, AI: 259, O: 42	USA	GPL17586	SLE samples from patients enrolled in a clinical trial
