# Supplementary material for: SLE-diseaseome: a comprehensive meta-collection of systemic lupus erythematosus relevant functional pathways
Source: Bioinform Adv. 2026 Feb 18;6(1):vbag061. doi: 10.1093/bioadv/vbag061 (PMC12989159; doi:10.1093/bioadv/vbag061)
Supplement: vbag061_Supplementary_Data [file vbag061_supplementary_data.zip › SupplementaryTable2.pdf]

Supplementary Table 2: Information about gene signatures and functional databases. The table contains the original source of each signature set used, whether it be a scientific article, an R package, a curated database, as well as the version used. The Database/Collection column contains specific identifiers for each signature set. Additionally, the table includes the original gene set number, the average pathway length (and their standard deviation) and a brief description of each collection.

Source	Version	Database/ Collection	Number of pathways	Average pathway length (standard deviation)	Description
Gene Ontology	GO release (2025-07)	Biological Process (BP)	12695	12.41 (±49.56)	Curated functional annotations
		Cellular Component (CC)	1842	58.19 (±398.8)	Curated functional annotations
		Molecular Function (MF)	4541	19.73 (±221.61)	Curated functional annotations
KEGG	Release 115 (2025-07)	KEGG	345	101.79 (±111.51)	Curated signaling and metabolic pathways
Reactome	Release 92 (2025-03)	Reactome	2501	47.95 (±135.62)	Expert-curated biological pathways
BloodGen3Modules R package	1.16	B3M	382	37.09 (±27.44)	Immune related co-expressed gene modules
tmod R package	0.50.13	tmod (LI)	346	21.06 (±30.61)	Immune related co-expressed gene modules from article PMC3946932
		tmod (DC)	260	50.58 (±48.11)	Immune related co-expressed gene modules from article PMC2727981
Wikipathways	Release 2025-06	Wikipathways	703	43.37 (±52.04)	Community-curated biological pathways
PMC5688663	xCell 2.0	xCell	46	153.63 (±113.01)	Blood-derived cell type gene signatures
PMC11291291	Cited by PMID/PMC	Literature	4	6 (±2.16)	NK subtypes signatures (single cell)
PMC8012727	Cited by PMID/PMC	Literature	6	5.33 (±3.83)	B cell subtypes signatures (single cell)
PMC11148857	Cited by PMID/PMC	Literature	5	499 (±324.79)	IFN-subtypes signatures
