# Supplementary material for: SLE-diseaseome: a comprehensive meta-collection of systemic lupus erythematosus relevant functional pathways
Source: Bioinform Adv. 2026 Feb 18;6(1):vbag061. doi: 10.1093/bioadv/vbag061 (PMC12989159; doi:10.1093/bioadv/vbag061)
Supplement: vbag061_Supplementary_Data [file vbag061_supplementary_data.zip › SupplementaryTable3.pdf]

Supplementary Table 3: Number of signatures from each source during the different steps. The table contains the original number of signatures from each database / collection and how changes in number (and in percentage comparing with the previous steps each time). GO: Gene Ontology; BP: Biological Process; CC: Celular Component; MF: Molecular Function; B3M: database from BloodGen3Modules R package. DRGs: Disease-relevant functional gene sets.

Database/ Collection	Original pathways	Split pathways (>3 genes)	Filtering by Set packing	Filtering by Jaccard index	DRGs
GO BP	12695	15321 (120,69%)	15122 (98,7%)	14983 (99,08%)	1713 (11,43%)
GO CC	1842	3555 (193%)	3491 (98,2%)	3452 (98,88%)	422 (12,22%)
GO MF	4541	4870 (107,25%)	4719 (96,9%)	4480 (94,94%)	533 (11,9%)
KEGG	345	1976 (572,75%)	1931 (97,72%)	1919 (99,38%)	293 (15,27%)
Reactome	2501	7011 (280,33%)	6247 (89,1%)	6009 (96,19%)	920 (15,31%)
B3M	382	1309 (342,67%)	1309 (100%)	1309 (100%)	325 (24,83%)
tmod	606	1862 (307,26%)	1855 (99,62%)	1855 (100%)	494 (26,63%)
Wikipathways	703	2553 (363,16%)	2526 (98,94%)	2510 (99,37%)	433 (17,25%)
xCell	46	292 (634,78%)	290 (99,32%)	289 (99,66%)	61 (21,11%)
Literature	15	48 (320%)	47 (97,92%)	47 (100%)	18 (38,3%)
