# Supplementary material for: SLE-diseaseome: a comprehensive meta-collection of systemic lupus erythematosus relevant functional pathways
Source: Bioinform Adv. 2026 Feb 18;6(1):vbag061. doi: 10.1093/bioadv/vbag061 (PMC12989159; doi:10.1093/bioadv/vbag061)
Supplement: vbag061_Supplementary_Data [file vbag061_supplementary_data.zip › Supplementary File 1.pdf]

1. disectDB function (pathMED)

minSplitSize: numeric, minimum number of genes in a subpathway.

minPathSize: numeric, minimum number of genes in a pathway to consider splitting it.

maxSplits: numeric, maximum number of subpathways derived from the original pathway. If NULL (default), there is not limit.

explainedVariance: numeric, percentage of cumulative variance explained within a pathway. This parameter is used to select the number of subdivisions of a pathway that manages to explain at least the percentage of variance defined by explainedVariance.

percSharedGenes: numeric, minimum percentage of common genes across datasets to merge or not the different datasets before clustering. If NULL or this percentage is not reached, clustering is performed for each dataset independently and consensus subpathways are obtained from co-occurrence clustering across datasets. Otherwise, the datasets can be joined into a single matrix where the subpathway clustering can be performed directly.

Rationale / main assumptions: The minimum threshold of 8 genes was determined based on our internal test. Initially, we considered that a biological pathway must contain at least 3 genes to be meaningfully analyzed, as many single sample scoring and enrichment methods require a minimum of 3 features to compute reliable statistics and avoid results being driven by single genes. Under this assumption, the minimum value for minPathSize must be at least 6 to allow the algorithm to return sub-pathways containing at least 3 genes each.

We therefore initiated our analysis with minPathSize = 6 and progressively increased this value to evaluate its impact on the resulting pathway clusters. During this evaluation, we had two observations. First, the Gene Ontology database contains a large number of relatively small pathways, primarily in the range of 6 to 10 genes, followed by Reactome and Wikipathways, to a lesser extent. Secondly, setting the threshold as 6 as opposed to 8 resulted in approximately 1,500 additional pathways that would be analyzed for splitting. However, meaningful clusters with at least 3 genes only started to appear when minPathSize was larger than 8. Setting the threshold as 6, 7 or 8 did not yield meaningful clustering results, potentially because either they already represented well-defined biological modules, or any potential split would result in clusters with less than 3 genes, which would not be applicable to scoring.

Based on these observations, a threshold of 8 genes was selected to preserve small but biologically meaningful pathways, while enabling splits of large and more heterogeneous pathways.

The parameters maxSplit and percSharedGenes were set to NULL. This choice allows pathways to be subdivided without imposing an upper limit on the number of resulting sub-pathways (**maxSplit = NULL**) and ensures that pathway subdivision is performed independently within each dataset (**percSharedGenes = NULL**). This design enables a subsequent co-occurrence clustering step, in which only pathway clusters that are consistently observed across multiple studies are retained.

Finally, the parameter **explainedVariance** was empirically set to 70. During the development of the *pathMED* R package, we observed that higher values of explainedVariance tended to increase the number of clusters in certain datasets, particularly those containing repeated or longitudinal samples from the same individuals. In such cases, a very high proportion of variance explained may reflect patient-specific variation rather than reliably capturing underlying biological signals. These patient-dependent clusters are typically less reproducible across studies and are therefore removed during the co-occurrence analysis. Given that the present study integrates a large number of heterogeneous datasets, we selected a relatively high explainedVariance value to preserve potentially relevant sub-patterns within pathways, while avoiding excessive sensitivity to individual-level effects. For other applications, this parameter may be further refined empirically and can be adapted according to the characteristics of the data, such as sample size, patient homogeneity, or the presence of longitudinal measurements.

2. Set Packing (setPackingFilter function - Github)

coverage.thr: numeric, minimum proportion of genes in a gene set G, that must be jointly covered by a selected combination of smaller gene sets gs $\{g_1, g_2, \ldots, g_n\}$

gainIndex: numeric, minimum required difference (in percentage) in the proportion of patients showing significant dysregulation between G and the selected combination of gene sets gs, used to decide whether the large gene set or the smaller gene sets retain more disease-relevant information.

minCorr: numeric, minimum average Pearson correlation coefficient between M-scores of G and the selected gene set combination gs across datasets, used to assess whether the smaller gene sets capture variability beyond that represented by G.

max.combs: numeric, maximum number of gene sets gs that can be combined to attempt to reconstruct a larger gene set G.

<u>**Rationale / main assumptions**</u>: The set packing procedure first identifies, for each gene set G, all smaller gene sets $gs\,\{g_1, g_2, \dots, g_n\}$ that are strict subsets of G. An optimal combination of these smaller gene sets is then selected to maximize their joint coverage of the genes in G. The parameter coverage.thr controls the stringency of this step. A **coverage.tr=0.8** means that the combination of gene sets gs must cover at least the 80 percent of the genes from G. Higher values enforce stricter reconstruction of G by the smaller gene sets, In practice, it would result in fewer effective set packing events and potentially higher redundancy. In contrast, lower values promote more aggressive packing, reducing the total number of gene sets while allowing weaker compositional relationships. The selected value represents a tradeoff between redundancy reduction and preservation of biologically coherent pathways.

In addition, and optionally, two complementary criteria can be used to determine whether the original gene set G or the selected smaller gene sets should be retained. First, the average Pearson's correlation comparing the dysregulation scores (i.e., M-scores) between G and gs is evaluated across patient samples. The minCorr parameter ensures that when correlations fall below the specified threshold (**minCorr = 0.75**), the smaller gene sets are preferentially retained, as it indicates that they can capture additional axes of biological variability not represented by G. Increasing minCorr therefore biases the procedure toward retaining the gene sets gs. Second, information gain was defined as the average difference in the proportion of patients exhibiting significant gene-set dysregulation (|M-score| ≥ 1.65) between G and the selected gene set combination gs across datasets. The gainIndex parameter quantifies whether the smaller gene sets provide increased sensitivity for detecting disease-associated perturbations. The chosen **gainIndex = 10** is intentionally restrictive: if the smaller gene sets identify at least 10% more patients with significant dysregulation than , they are retained to replace G; otherwise G is retained. Higher gainIndex values would reduce set packing and maintain redundancy, whereas lower values would be overly permissive and could yield results similar to omitting this filter. These two parameters (minCorr and gainIndex), although optional, can provide more biological context to ensure the most meaningful set packing application.

Finally, **max.combs** defines the maximum number of gene sets that can be combined to approximate G. This value was primarily constrained by computational feasibility (due the number of combinations increases on a factorial scale). Empirically, **max.combs = 8** was sufficient in the majority of cases to achieve the highest coverage.

3. Filter based on Jaccard Similarity (getNodes function – Github)

simmilarity.threshold: numeric, minimum Jaccard index required to consider two gene sets as highly similar or potentially redundant.

Rationale / main assumptions: In general, Jaccard index values greater than 0.7 are typically interpreted as indicating high similarity and near-redundancy between gene sets. In this study, we adopted a more restrictive threshold to ensure that only strongly overlapping pathways were collapsed. Specifically, gene set pairs with a Jaccard index greater than 0.8 were considered redundant (**simmilarity.threshold = 0.8**). This stringent cutoff minimizes the risk of merging gene sets that share only partial biological overlaps and helps preserve distinct functional signals. However, for very small gene sets (with ≤ 4 genes), high Jaccard similarity values can only be achieved at specific discrete levels (e.g., 3 out of 4 shared genes correspond to a Jaccard index of 0.75). To enable meaningful similarity assessment in these cases, a slightly relaxed threshold (> 0.7) was applied for gene sets containing 4 or fewer genes. This exception allows the identification of near-identical small pathways.

Increasing simmilarity.threshold would result in fewer gene sets being merged, thus retaining greater redundancy, whereas lowering it would risk collapsing gene sets with limited overlap, potentially creating looser and less biologically coherent functional groups.
