# Supplementary figures and images for: SLE-diseaseome: a comprehensive meta-collection of systemic lupus erythematosus relevant functional pathways
Source: Bioinform Adv. 2026 Feb 18;6(1):vbag061. doi: 10.1093/bioadv/vbag061 (PMC12989159; doi:10.1093/bioadv/vbag061)

**A****Random-based FPR**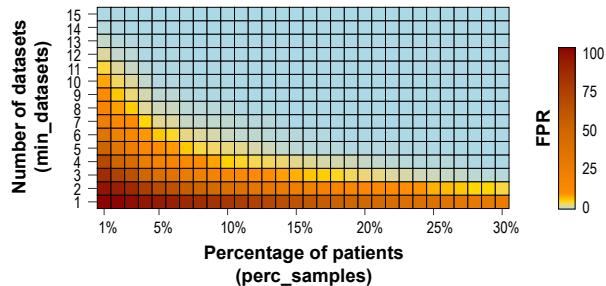**B****Retained information**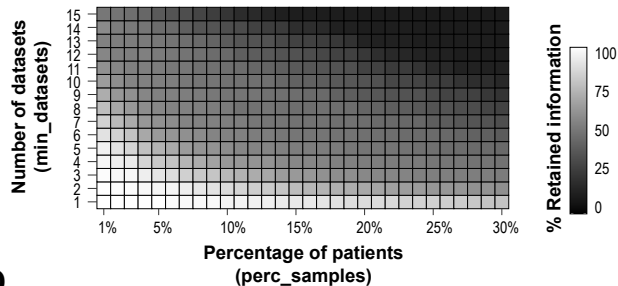**C****Shannon index**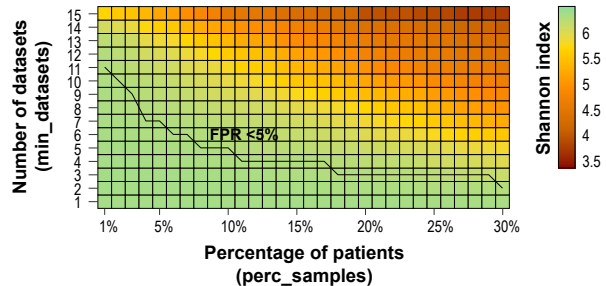**D****Number of DRGs**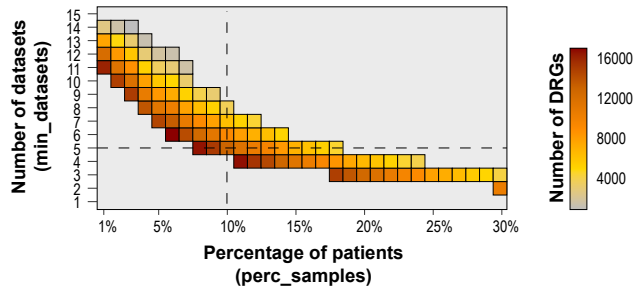

Supplement: vbag061_Supplementary_Data [file vbag061_supplementary_data.zip › SupplementaryFigure1.pdf]

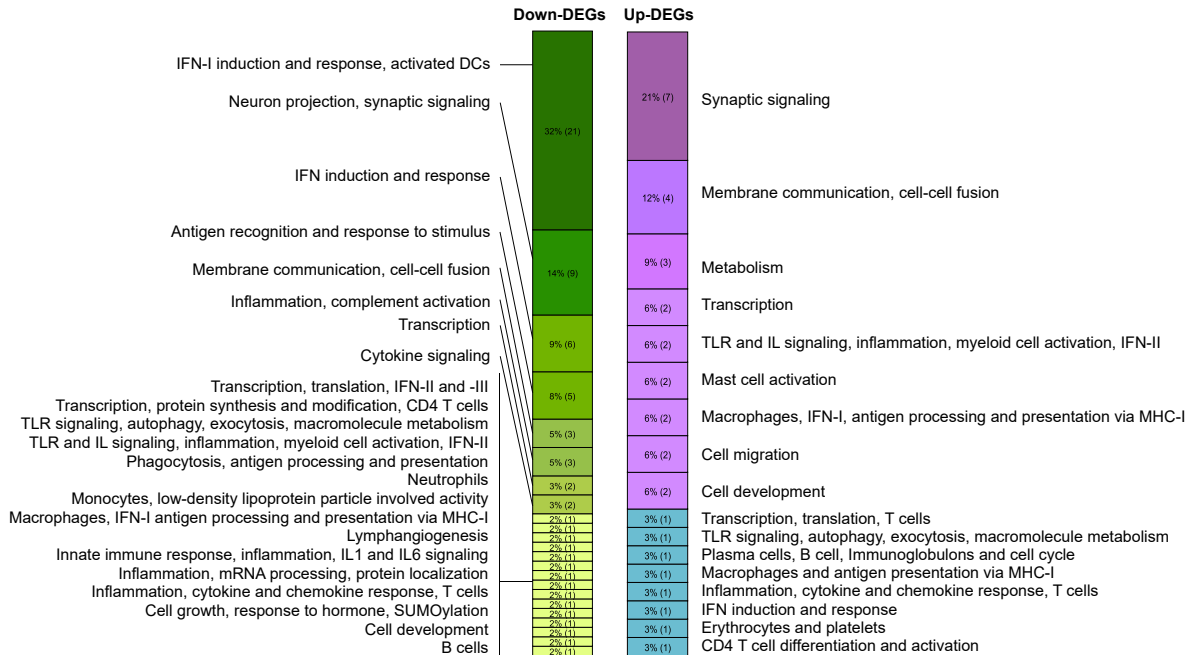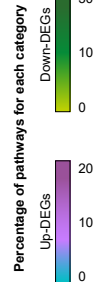

Supplement: vbag061_Supplementary_Data [file vbag061_supplementary_data.zip › SupplementaryFigure2.pdf]
